# Prognostic Value of Prognostic Nutritional Index in Patients With Colorectal Cancer Undergoing Surgical Treatment

**DOI:** 10.3389/fnut.2022.794489

**Published:** 2022-03-11

**Authors:** Hailun Xie, Lishuang Wei, Guanghui Yuan, Mingxiang Liu, Shuangyi Tang, Jialiang Gan

**Affiliations:** ^1^Department of Colorectal and Anal Surgery, The First Affiliated Hospital, Guangxi Medical University, Nanning, China; ^2^Department of Respiratory Medicine, The First Affiliated Hospital, Guangxi Medical University, Nanning, China; ^3^Department of Pharmacy, The First Affiliated Hospital, Guangxi Medical University, Nanning, China; ^4^Guangxi Key Laboratory of Enhanced Recovery After Surgery for Gastrointestinal Cancer, Nanning, China

**Keywords:** prognostic nutritional index, colorectal cancer, complication, prognosis, nutrition

## Abstract

**Background:**

To investigate the relationship between prognostic nutritional index (PNI) and the survival of patients with colorectal cancer (CRC) undergoing surgical treatment.

**Methods:**

In total 1,014 CRC patients who underwent surgical treatment were enrolled. Logistic regression analysis was used to identify the features that influenced postoperative complications in CRC patients. Restricted cubic spline was used to assess the dose-response relationship between PNI and survival in CRC patients. Kaplan-Meier method and log-rank test were used to compare survival differences between groups of CRC patients. Cox proportional risk regression models was used to assess independent risk factors for progression-free survival (PFS) and overall survival (OS) of CRC patients.

**Results:**

Low PNI was associated with high tumor burden, invasive pathological features, and poor host status. Compared with patients with high PNI, patients with low PNI have a higher incidence of complications and longer hospital stay. Low PNI was an independent risk factor for postoperative complications in CRC patients. for every SD increased in PNI, the risk of poor prognosis for CRC patients was reduced by 2.3% (HR = 0.977, 95%CI = 0.962–0.993, *p* = 0.004) in PFS, and 2.3% (HR = 0.977, 95%CI = 0.962–0.993, *p* = 0.004) in OS. PNI was an independent prognostic factor affecting the PFS and OS of CRC patients. Finally, we constructed the PNI-based nomograms to predict postoperative complications, 1–5 years PFS and OS in CRC patients. Concordance index and calibration curve indicated that the PNI-based nomograms have moderate prediction accuracy.

**Conclusion:**

PNI is an independent risk factor affecting postoperative complications, PFS and OS of CRC patients, and is a useful supplement to the TNM stage.

## Introduction

Colorectal cancer (CRC) is one of the most common gastrointestinal malignancy in the world, ranking third in incidence and second in cancer-related death, according to the latest data. It is estimated that more than 1.93 million new CRC cases and 935,000 death occur globally in 2020, accounting for ~10% of new cancer cases and cancer-related death worldwide (1). In China, CRC is the fourth most common malignancy (~388,000 cases) and the fifth most common cause of cancer-related death (~187,000 cases) (2). With the development of treatment methods such as surgery, radiochemotherapy, immunotherapy, and targeted therapy, the 5- and 10- year survival rate of patients with early CRC can reach 58–65%, but the survival rate of CRC patients with recurrence and metastasis can be reduce to 10% (3). Microvascular invasion, tumor-related factors, tumor-node-metastasis (TNM) stage, microsatellite status, etc. have been reported as prognostic factors for CRC patients. However, effective prognostic factors are still lacking, especially simple and economical biomarkers. Therefore, there is an urgent need to find effective prognostic indicators to help clinicians adopt optimal prevention and treatment strategies to reduce CRC-related mortality.

More and more evidences indicate that the gradual decline of nutritional status is related to disease progression and is one of the main reasons for poor treatment effectiveness. Perioperative malnutrition not only significantly increases the incidence of postoperative complications, but is also associated with poor long-term outcomes (4, 5). In addition, the immune status is also an important factor affecting the clinical outcome of patients (6). Various prognostic indicators calculated from serum parameters have been confirmed to be associated with prognosis of patients with cancer. The evaluation of preoperative immune-nutritional status can predict the risk and survival rate of surgery, which is helpful to determine strategies to prevent postoperative complications and improve the prognosis. The prognostic nutritional index (PNI), which combines nutritional and immune parameters, has been proven to be a good predictor of postoperative complications and survival rates for many malignancy (7–9). Tokunaga et al. (8) conducted a study of 556 cases of CRC in 2015. The results indicated that preoperative PNI could effectively predict severe complications, recurrence and poor prognosis in CRC patients undergoing resection. Noh et al. (10) found that low PNI was associated with increased postoperative complications, long hospital stays, poor prognosis, and aggressive tumor phenotype. A meta-analysis in 2019 also showed that compared with CRC patients with low PNI, the overall survival (OS) of those with high PNI was significantly improved (11).

There are still few studies on the relationship between PNI and postoperative complications and long-term prognosis in CRC patients. In addition, the prognostic prediction efficiency of a single indicator is still low, and the combination of multiple indicators to construct a nomogram may be an effective means to improve the prognostic predictive performance. Therefore, this study aimed to explore the value of PNI in evaluating postoperative complications and long-term prognosis of CRC patients undergoing surgical treatment, and construct PNI-based nomograms to individually predict the prognosis of CRC patients, so as to provide certain guidance for formulating treatment strategies for CRC patients.

## Patients and Methods

### Study Design

This study included CRC patients who underwent surgical treatment at the department of colorectal and anal Surgery of the First Affiliated Hospital of Guangxi Medical University from January 2012 to December 2015. Inclusion criteria are as follows: (1) Histopathologically confirmed colon or rectal cancer; (2) The primary tumor received surgical resection; 3) Complete available clinicopathological data; (4) Complete postoperative follow-up data. The exclusion criteria were as follows: (1) Patients complicated with other primary malignancy during the same period; (2) Patients with autoimmune diseases, blood diseases, obviously abnormal liver function or abnormal kidney function; (3) Patients with obvious clinical evidence of infection or inflammation; (4) Patients who lost follow-up or did not have complete data. This study strictly complied with the Helsinki Declaration during the research process, and was approved by the Ethics Committee of the First Affiliated Hospital of Guangxi Medical University, with the approval number: 2021 (KY-E-043).

### Data Collection

Clinicopathological features included the following aspects: basic information included sex, age, height, and weight; preoperative basic diseases included hypertension and diabetes; preoperative laboratory serological tests included neutrophil count, lymphocyte count, and albumin (hypoproteinaemia, defined as albumin <35 g/L) and serum CEA level (normal, <5.00 ng/ml; high, ≥5.00 ng/ml), All preoperative laboratory serological tests were performed 1 week before surgery; Pathological characteristics included TNM stage, pathological tumor infiltration depth (pT) stage, pathological lymph node metastasis (pN stage), distant metastasis, perineural invasion, vascular invasion, pathological type, differentiation, tumor location, and tumor size. Surgical information included surgical approach (laparoscopic or open), operating time (median 192 min), and intraoperative bleeding (median 100 mL). Body mass index (BMI) was defined as weight (kg) / square height (m^2^) (low: <18.5, normal: 18.5–24, high: ≥24). PNI was defined as: serum albumin (g/L) + 5 × total peripheral lymphocyte count (×10^9^/L). Neutrophil to lymphocyte ratio (NLR) was defined as: neutrophil count (10^9^/L) / lymphocyte count (10^9^/L). Platelet to lymphocyte ratio (PLR) was defined as: platelet count (10^9^/L) / lymphocyte count (10^9^/L). The postoperative complications of CRC patients in this study were strictly classified according to the modified Clavien complication classification system (12, 13).

### Follow-Up

CRC patients were followed up every 3 months for 2 years after surgery, and every 6 months thereafter. The last follow-up date was February 04, 2021. Progression-free survival (PFS) was defined as the time interval between the date of surgery and the patient's disease recurrence, death, or the last follow-up; OS was defined as the time interval between the date of surgery and the patient's death or last follow-up.

### Statistical Analysis

Continuous data was presented as means with standard deviations (SDs), and classification data was presented as frequencies and percentages. Chi-square test or *t*-test was used to analyze the correlation between PNI and various clinicopathological features. The optimal cutoff value of PNI was determined by the standardized log-rank statistic (R package “survminer”) based on the OS. Logistic regression analysis was used to identify the features that influenced postoperative complications in CRC patients. Restricted cubic spline (RCS) was used to assess the dose-response relationship between PNI and survival in CRC patients. Kaplan-Meier method and log-rank test were used to compare survival differences between groups of CRC patients. Cox proportional risk regression models was used to assess independent risk factors for PFS and OS of CRC patients. Receiver operator characteristic curve (ROC) analysis was used to compare the effectiveness of PNI and other prognostic indicators in predicting PFS and OS. In addition, based on the results of multivariate analysis, we constructed the PNI-based nomograms to predict postoperative complications, 1–5 years PFS and OS in CRC patients. Concordance index (C-index) and calibration curve were used to assess the prognostic accuracy of PNI-based nomograms. Finally, time-dependent ROC and decision curve analysis (DCA) were used to compare the ability of the nomogram with the traditional TNM stage in predicting long-term prognosis of CRC patients. A *p* < 0.05 was considered statistically significant. All statistical analysis was performed using SPSS 24.0 (IBMSPSS, IBM CorPoration, Armonk, NY) and R software (3.5.3; http://www.r-Project.org).

## Results

### Characteristics of Clinical Baseline

A total of 1,014 CRC patients were enrolled in this study. The optimal cut-off value of PNI was determined as 44.65 by the standardized log-rank statistic (Supplementary Figure S1). Based on this cut-off value, there were 334 (38.9%) patients in the low PNI group and 680 (61.1%) patients in the high PNI group. The characteristics of CRC patients were presented in Table 1. There were 639 (63.0%) males and 375 (37.0%) females. 532 (52.5%) patients were <60 years old, and 482(47.5%) patients were ≥ 60 years old, with an average age of 57.33 ± 13.34. There were 504 (56.1%) cases of rectal cancer and 510 (43.9%) cases of colon cancer. There were 184 (18.1%) TNM stage I, 328 (32.3%) TNM stage II, 397 (38.7%) TNM stage III, and 105 (10.8%) TNM stage IV. The median follow-up time of 67.2 months (1–100.9 months).

**Table 1:** The relationships between the PNI and clinicopathological factors of CRC patients.

**Features**	**Total (*n* = 1,014)**	**PNI**		**X^**2**^/t**	***P* value**
		**Low (*n* = 334)**	**High (*n* = 680)**		
Gender (Male)	639 (63.0%)	224 (67.1%)	415 (61.0%)	3.502	0.061
Age (Years) (≥60)	482 (47.5%)	197 (59.0%)	285 (41.9%)	26.171	<0.001
Age (Years)	57.33 ± 13.338	59.27 ± 14.219	56.11 ± 12.605	3.700	<0.001
BMI				25.599	<0.001
Low	138 (13.6%)	69 (20.7%)	69 (10.1%)		
Normal	599 (59.1%)	195 (58.4%)	404 (59.4%)		
High	277 (27.3%)	70 (21.0%)	207 (30.4%)		
Hypertension (Yes)	153 (15.1%)	64 (19.2%)	89 (13.1%)	6.449	0.011
Diabetes (Yes)	65 (6.4%)	31 (9.3%)	34 (5.0%)	6.844	0.009
pT stage (T3-4)	765 (75.4%)	250 (74.9%)	515 (75.7%)	0.095	0.758
pN stage				2.439	0.295
N0	551 (54.1%)	191 (57.2%)	358 (52.6%)		
N1	295 (29.1%)	87 (26.0%)	208 (30.6%)		
N2	170 (16.8%)	56 (16.8%)	114 (16.8%)		
Clinical distant metastasis (Yes)	105 (10.8%)	47 (14.1%)	58 (8.5%)	7.412	0.006
TNM stage				10.826	0.013
I stage	184 (18.1%)	68 (20.4%)	116 (17.1%)		
II stage	328 (32.3%)	103 (30.8%)	225 (33.1%)		
III stage	397 (39.2%)	116 (34.7%)	281 (41.3%)		
IV stage	105 (10.4%)	47 (14.1%)	58 (8.5%)		
Tumor location (Colon)	510 (50.3%)	191 (57.2%)	319 (46.9%)	9.457	0.002
Tumor size (≥5 cm)	492 (48.5%)	205 (61.4%)	287 (42.2%)	32.958	<0.001
Perineural invasion (Positive)	90 (8.9%)	32 (9.6%)	58 (8.5%)	0.306	0.580
Vascular invasion (Positive)	151 (14.9%)	47 (14.1%)	104 (15.3%)	0.264	0.607
Macroscopic type				6.885	0.032
Protrude type	250 (24.7%)	94 (28.1%)	156 (22.9%)		
Infiltrating type	95 (9.4%)	38 (11.4%)	57 (8.4%)		
Ulcerative type	669 (66.0%)	202 (60.5%)	467 (68.7%)		
Histological type (Poor)	123 (12.1%)	40 (12.0%)	83 (12.2%)	0.011	0.916
CEA (≥5 ng/ml)	428 (42.2%)	169 (50.6%)	259 (38.1%)	14.372	<0.001
Length of stay	18.00 (16.00, 22.00)	20.00 (17.00, 24.00)	18.00 (16.00, 21.00)	3.854	<0.001
Recurrence and metastasis (Yes)	297 (29.3)	118 (35.3)	179 (26.3)	8.771	0.003
Death (Yes)	444 (43.8)	182 (54.5)	262 (38.5)	23.184	<0.001

*CRC, colorectal cancer; BMI, body mass index; PNI, prognostic nutrition index*.

### Correlation Analysis of PNI and Various Clinical Characteristics

We conducted a correlation analysis between PNI and clinicopathological features. The results showed that low PNI was associated with advanced age, low BMI, preoperative comorbidity (hypertension, diabetes), distant metastasis, advanced TNM stage, colon cancer, large tumor size, macroscopic type, high CEA level, long hospital stay, high recurrence and high mortality (All *p* < 0.05). While there were no statistically significant differences between the high and low PNI groups in terms of gender, pT stage, pN stage, perineural invasion, vascular invasion, differentiation and other tumor-related factors (Table 1 and Supplementary Figure S2).

### Relationship Between PNI and Postoperative Complications in CRC Patients

Among 1,014 CRC patients, a total of 180 (17.8%) patients had various postoperative complications, including 18 (1.78%) cases of intestinal obstruction, 30 (2.96%) cases of anastomotic problems, 54 (5.33%) cases of wound problems, 29 (2.86%) cases of pulmonary infection, 13 (1.28%) cases of gastrointestinal problems, 6 (0.59%) cases of abdominal infection and 30 (2.96%) cases of other complications. According to the modified Clavien complication classification system, there are 64 (6.3%) grade I complications, 89 (8.8%) grade II complications, and 18 (1.8%) grade III complications, including grade IIIa 10 (1.0%) cases, 8 (0.8%) cases of grade IIIb complications, 8 (0.8%) cases of grade IV complications, including 5 (0.5%) cases of grade IVa, 3 (0.3%) cases of grade IVb complications, and 1 (0.1%) complication of grade V complications. Compared with those with high PNI, the total postoperative complications (X^2^ = 15.771, *p* < 0.001) of patients with low PNI significantly increased, especially grade I (X^2^ = 16.074, *p* < 0.001) and grade III (X^2^ = 4.244, *p* < 0.001) (Supplementary Table S1). The complication rate in the PNI (Q1) group was 6.61%, while the complication rate in the PNI (Q4) group was 3.45%. In addition, the length of hospital stays gradually decreased from 20 days for patients with PNI <43.40 to 17 days for patients with PNI > 50.70 (Supplementary Figure S3). Univariate logistic regression analysis showed that age, hypertension, PNI, surgical approach, operating time, intraoperative bleeding and serum CEA levels were associated with postoperative complications; However, multivariate analysis showed that only age (≥60 years) (OR: 1.677, 95%CI: 1.181–2.380, *p* = 0.004) and low PNI (HR: 1.580, 95%CI: 1.122–2.27, *p* = 0.009), Operating time (≥192 min) (OR: 1.530, 95%CI: 1.104–2.122, *p* = 0.044) and intraoperative bleeding (≥100 mL) (OR: 1.660, 95% CI: 1.109–2.484, *p* = 0.014) were independent risk factors for postoperative complications in CRC patients (Supplementary Table S2).

### Relationship Between PNI and Survival in CRC Patients

**Figure 1:**
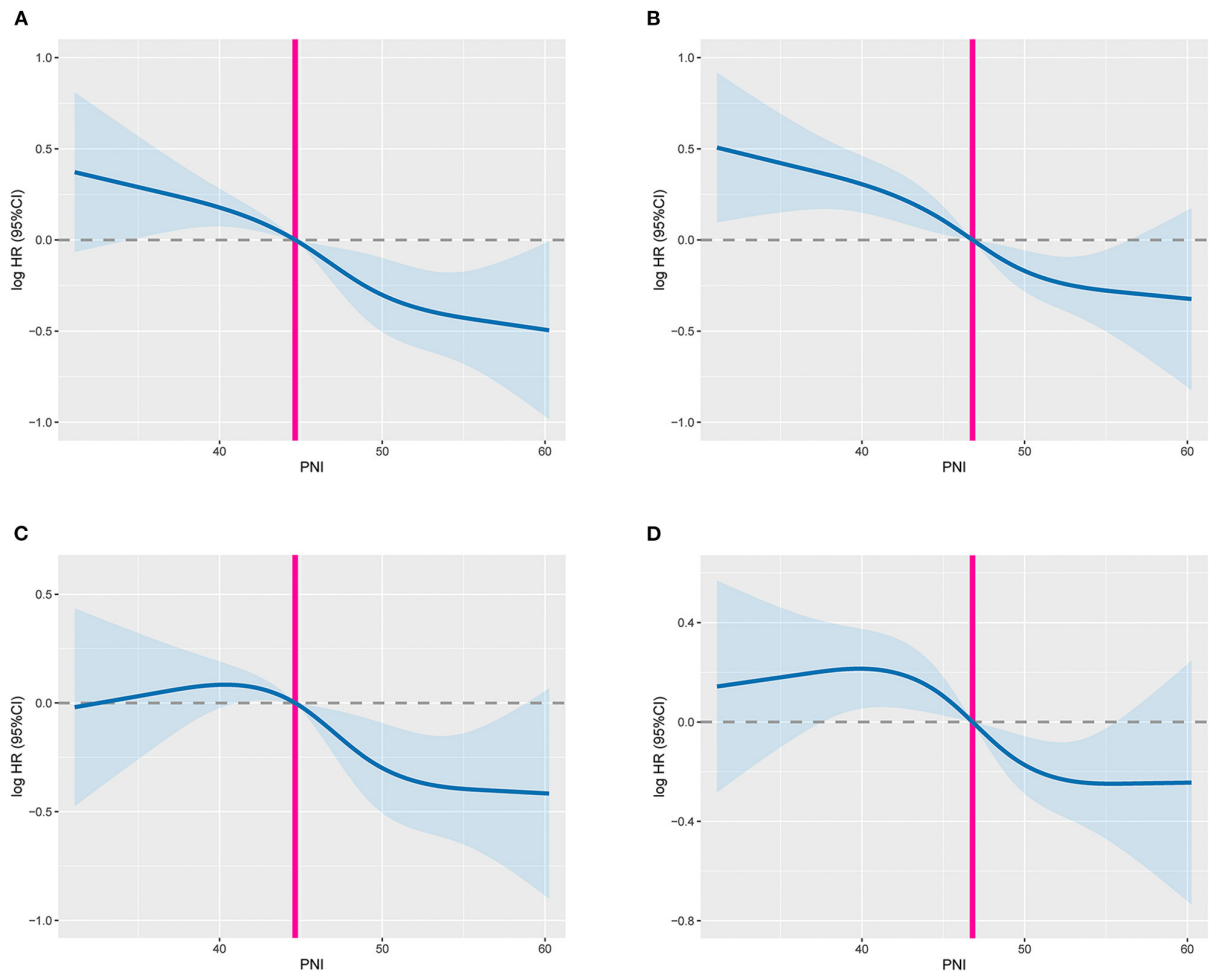
The dose-response relationship between PNI and survival in CRC patients. **(A)** Unadjusted RCS of PFS; **(B)** unadjusted RCS of OS; **(C)** adjusted RCS of PFS; **(D)** adjusted RCS of OS; The model adjusted for gender, age, BMI, hypertension, diabetes, pT stage, pN stage, clinical distant metastasis, tumor location, tumor size, perineural invasion, vascular invasion, macroscopic type, and histological type.

**Figure 2:**
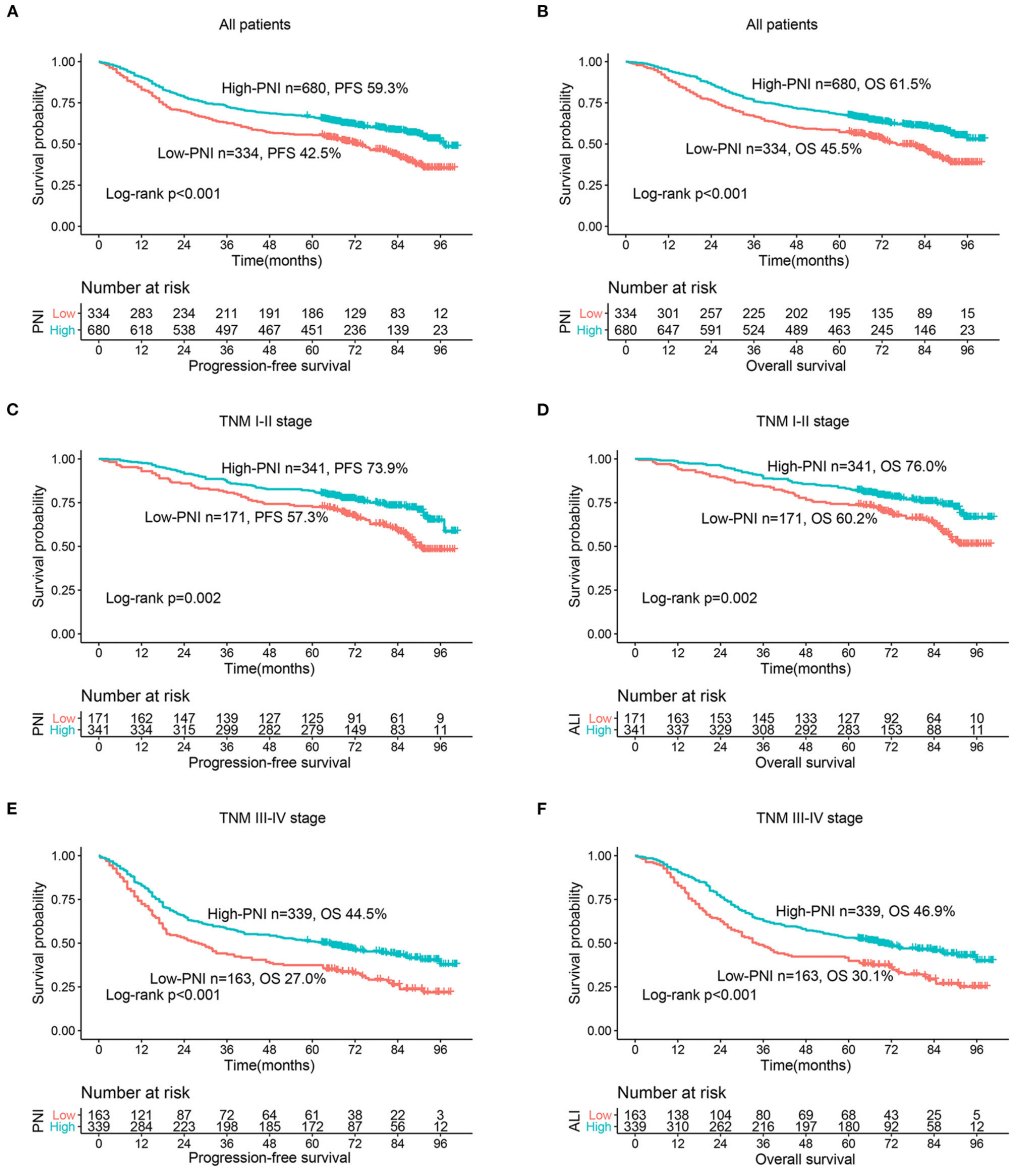
Kaplan-Meier curves of different PNI groups in CRC patients. **(A)** PFS of PNI; **(B)** OS of PNI; **(C)** PFS of PNI based on TNM I-II stage; **(D)** OS of PNI based on TNM I-II stage; **(E)** PFS of PNI based on TNM III-IV stage; **(F)** OS of PNI based on TNM III-IV stage.

The RCS showed that with the increase of PNI, The PFS (Figures 1A,C) and OS (Figures 1B,D) of CRC patients increased gradually. After adjusting confounding factors, there was still a negative non-linear relationship between PNI and survival of CRC patients. During follow-up, a total of 297 (29.3%) patients had recurrence and metastasis, including 118 patients in the low PNI group (35.33% of the low PNI group) and 179 patients in the high PNI group (26.32% of the high PNI group). The PFS of the low PNI group was significantly lower than that of the high PNI group (42.5 vs. 59.3%, *p* < 0.001) (Figure 2A). By the last follow-up, 444 (43.79%) patients died, including 182 patients in the low PNI group (54.49% of the low PNI group) and 262 patients in the high PNI group (38.53% of the high PNI group). The OS of patients with low PNI was significantly lower than that of the patients with high PNI (45.5 vs. 61.5%, *p* < 0.001) (Figure 2B). In addition, stratified analysis showed that for patients with stage I-II CRC, PFS (57.3 vs. 73.9%, *p* =0.002) and OS (60.2 vs. 76.0%, p = 0.002) in the low PNI group were significantly lower than those in the high PNI group (Figures 2C,D). Similarly, for patients with stage III-IV CRC, PFS (27.0 vs. 44.5%, *p* < 0.001) and OS (30.1 vs. 46.9%, *p* < 0.001) in the low PNI group were also significantly lower than those in the high PNI group (Figures 2E,F).

### Prognostic Factors Affecting PFS and OS in CRC Patients

In univariate analysis, PFS was affected by the following clinicopathological characteristics: Age (*p* = 0.002), BMI (*p* = 0.026), PNI (*p* < 0.001), pT stage (*p* < 0.001), pN stage (*p* < 0.001), distant metastasis (*p* < 0.001), tumor size (*p* = 0.021), perineural invasion (*p* < 0.001), vascular invasion (*p* < 0.001), differentiation (*p* = 0.010), surgical approach (*p* < 0.001), and CEA(*p* < 0.001). Subsequently, a multivariate analysis was performed on the 12 significant factors. The results showed that the independent prognostic factors affecting PFS in CRC patients were age (≥60 years) (HR = 1.297, 95%CI = 1.072–1.568, *p* = 0.007), PNI (HR = 1.359, 95%CI = 1.115–1.656, *p* = 0.002), pT stage (HR = 1.561, 95% CI = 1.184–2.059, *p* = 0.002), pN stage (*p* < 0.001), Distant metastasis (HR = 3.113, 95%CI = 2.420–4.005, *p* < 0.001), vascular invasion (HR = 1.363, 95%CI = 1.062–1.750, *p* = 0.015) and CEA (HR = 1.463, 95%CI = 1.203–1.780, *p* < 0.001) (Table 2).

**Table 2:** Univariate and multivariate Cox regression analysis of clinicopathological characteristics associated with progression-free survival.

**Characteristic**	**Progression-free survival**			
	**Univariate analysis**		**Multivariate analysis**	
	**HR (95%CI)**	***P* value**	**HR (95%CI)**	***P* value**
Gender (Female)	0.988 (0.819, 1.191)	0.895		
Age (≥60 years)	1.334 (1.113, 1.600)	0.002	1.297 (1.072, 1.568)	0.007
BMI		0.026		0.720
Low	Ref.		Ref.	
Normal	0.831 (0.647, 1.068)	0.148	0.964 (0.743, 1.250)	0.781
High	0.674 (0.504, 0.902)	0.008	0.891 (0.658, 1.207)	0.458
Hypertension (Yes)	1.155 (0.905, 1.475)	0.248		
Diabetes (Yes)	1.188 (0.838, 1.684)	0.334		
PNI (Low)	1.519 (1.264, 1.827)	<0.001	1.359 (1.115, 1.656)	0.002
pT stage (T3-4)	2.458 (1.894, 3.189)	<0.001	1.561 (1.184, 2.059)	0.002
pN stage		<0.001		<0.001
N0	Ref.		Ref.	
N1	1.648 (1.330, 2.042)	<0.001	1.405 (1.124, 1.755)	0.003
N2	3.620 (2.889, 4.534)	<0.001	2.645 (2.077, 3.370)	<0.001
Distant metastasis (Yes)	4.854 (3.869, 6.089)	<0.001	3.113 (2.420, 4.005)	<0.001
Tumor location (Colon)	0.969 (0.808, 1.161)	0.731		
Tumor size (≥5 cm)	1.238 (1.033, 1.484)	0.021	0.938 (0.775, 1.136)	0.514
Perineural invasion (Positive)	1.805 (1.379, 2.363)	<0.001	1.120 (0.829, 1.513)	0.461
Vascular invasion (Positive)	1.982 (1.592, 2.467)	<0.001	1.363 (1.062, 1.750)	0.015
Macroscopic type		0.095		
Protrude type	Ref.			
Infiltrating type	1.396 (0.991, 1.967)	0.057		
Ulcerative type	1.239 (0.988, 1.554)	0.063		
Histological grade (Poor)	1.409 (1.086, 1.829)	0.010	1.180 (0.898, 1.550)	0.235
Surgical approach (Open)	1.533 (1.278, 1.838)	<0.001	1.190 (0.979, 1.447)	0.080
Operating time (median) (≥192 min)	1.080 (0.900, 1.295)	0.408		
Blood loss (median) (≥100 mL)	1.143 (0.938, 1.394)	0.186		
CEA (≥5 ng/ml)	2.010 (1.676, 2.411)	<0.001	1.463 (1.203, 1.780)	<0.001
Postoperative chemoradiotherapy (Yes)	1.084 (0.903, 1.301)	0.389		

*CRC, colorectal cancer; BMI, body mass index; PNI, prognostic nutrition index*.

Similarly, univariate analysis showed that the following clinical features were significantly associated with OS: age (*p* < 0.002), BMI (*p* = 0.031), PNI (*p* < 0.001), pT stage (*p* < 0.001), pN stage (*p* < 0.001), distant metastasis (*p* < 0.001), tumor size (*p* = 0.002), perineural invasion (*p* < 0.001), vascular invasion (*p* < 0.001), differentiation (*p* = 0.002), surgical method (*p* < 0.001) and CEA (*p* < 0.001). However, in multivariate analysis, only age (≥60 years)(HR = 1.377, 95%CI = 1.132–1.674, *p* = 0.001), low PNI(HR = 1.329, 95%CI = 1.085–1.627, *p* = 0.006), high pT stage (T2-3) (HR = 1.599, 95% CI = 1.199–2.132, *p* = 0.001), high pN stage (*p* < 0.001), distant metastasis (HR = 3.325, 95%CI = 2.579–4.287, *p* < 0.001), vascular invasion (HR = 1.396, 95%CI = 1.083– 1.800, *p* = 0.010) and high CEA(HR = 1.445, 95%CI = 1.182–1.768, *p* < 0.001) were independent risk factors for OS in CRC patients (Table 3).

**Table 3:** Univariate and multivariate Cox regression analysis of clinicopathological characteristics associated with overall survival.

**Characteristic**	**Overall survival**			
	**Univariate analysis**		**Multivariate analysis**	
	**HR (95%CI)**	***P* value**	**HR (95%CI)**	***P* value**
Gender (Female)	0.994 (0.903, 1.095)	0.910		
Age (≥60 years)	1.421 (1.179, 1.713)	<0.001	1.377 (1.132, 1.674)	0.001
BMI		0.031		0.792
Low	Ref.		Ref.	
Normal	0.841 (0.650, 1.090)	0.190	0.976 (0.746, 1.276)	0.857
High	0.675 (0.500, 0.911)	0.010	0.908 (0.664, 1.243)	0.548
Hypertension (Yes)	1.172 (0.913, 1.504)	0.213		
Diabetes (Yes)	1.250 (0.881, 1.773)	0.212		
PNI (Low)	1.526 (1.263, 1.844)	<0.001	1.329 (1.085, 1.627)	0.006
pT stage (T3-4)	2.536 (1.931, 3.329)	<0.001	1.599 (1.199, 2.132)	0.001
pN stage		<0.001		<0.001
N0	Ref.		Ref.	
N1	1.627 (1.304, 2.030)	<0.001	1.376 (1.094, 1.730)	0.006
N2	3.651 (2.899, 4.598)	<0.001	2.588 (2.021, 3.315)	<0.001
Distant metastasis (Yes)	5.145 (4.092, 6.468)	<0.001	3.325 (2.579, 4.287)	<0.001
Tumor location (Colon)	0.997 (0.827, 1.200)	0.971		
Tumor size (≥5cm)	1.340 (1.112, 1.615)	0.002	1.023 (0.841, 1.246)	0.817
Perineural invasion (Positive)	1.789 (1.357, 2.359)	<0.001	1.079 (0.792, 1.468)	0.630
Vascular invasion (Positive)	2.042 (1.635, 2.551)	<0.001	1.396 (1.083, 1.800)	0.010
Macroscopic type		0.126		
Protrude type	Ref.			
Infiltrating type	1.349 (0.945, 1.927)	0.100		
Ulcerative type	1.248 (0.989, 1.576)	0.062		
Histological grade (Poor)	1.523 (1.173, 1.979)	0.002	1.292 (0.981, 1.701)	0.068
Surgical approach (Open)	1.616 (1.341, 1.949)	<0.001	1.216 (0.994, 1.487)	0.057
Operating time (median) (≥192 min)	1.087 (0.902, 1.311)	0.380		
Blood loss (median) (≥100 mL)	1.183 (0.964, 1.452)	0.108		
CEA (≥5 ng/ml)	2.025 (1.680, 2.441)	<0.001	1.445 (1.182, 1.768)	<0.001
Postoperative chemoradiotherapy (Yes)	1.004 (0.831, 1.212)	0.971		

*CRC, colorectal cancer; BMI, body mass index; PNI, prognostic nutrition index*.

The consistency test showed that when PNI was used as a continuous variable, for every SD increased in PNI, the risk of poor prognosis for CRC patients was reduced by 2.3% (HR = 0.977, 95%CI = 0.962–0.993, *p* = 0.004) in PFS, and 2.3% (HR = 0.977, 95%CI = 0.962–0.993, *p* = 0.004) in OS. In PFS, when PNI was split into quartiles, Q2, Q3, and Q4 were all positively associated with better prognosis (*p* < 0.001) with the lowest group (Q1) as a reference. After adjusting for confounders, the HRs of PFS were 0.768 (0.600, 0.984), 0.701 (0.543, 0.903), and 0.651 (0.499, 0.848), respectively (Supplementary Table S3, PFS). Also in OS, with the increase of PNI, the prognosis of patients gradually increased, and the HR of OS were 0.791 (0.615, 1.018), 0.674 (0.517, 0.877), and 0.666 (0.509, 0.873), respectively (Supplementary Table S3, OS). We performed a subgroup analysis using univariate Cox regression based on various clinical features. A total of 19 clinical features and 40 subgroups were included. The results showed that low PNI was a risk factor affecting the prognosis of CRC patients in most subgroups (Supplementary Figures S4A,B). In addition, we compared the effectiveness of PNI with other prognostic indicators (NLR and PLR) in predicting the clinical outcome of CRC patients through the ROC curve. The results showed that the ability of PNI was superior to other prognostic indicators in predicting PFS in CRC patients (Supplementary Figures S5A,B). Similarly, the ability of PNI was superior to NLR in predicting OS in CRC patients (Supplementary Figures S5C,D).

### Construction of PNI Based Prediction Model

**Figure 3:**
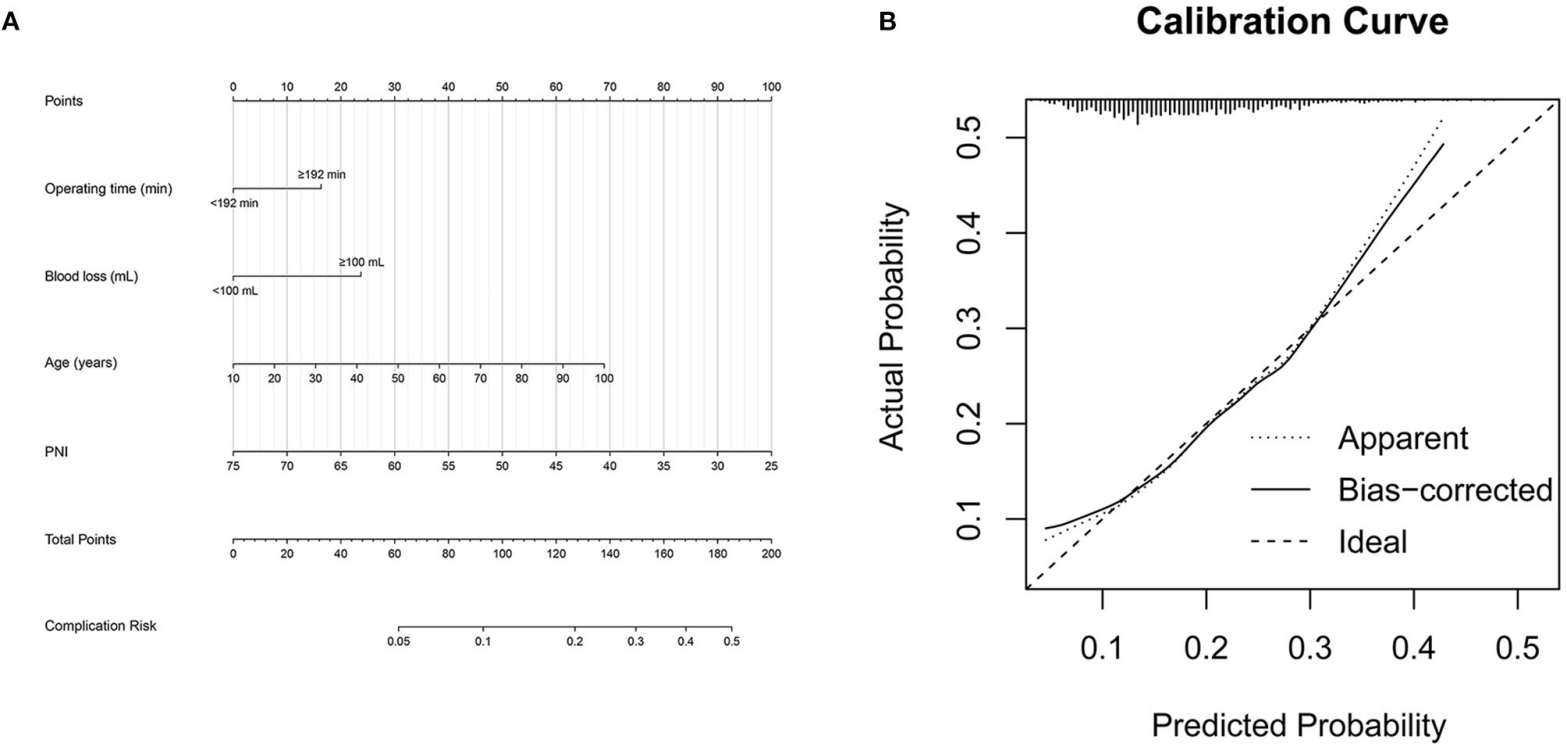
Construction a novel complication nomogram model. **(A)** Complication nomogram; **(B)** calibration curve of complication nomogram.

**Figure 4:**
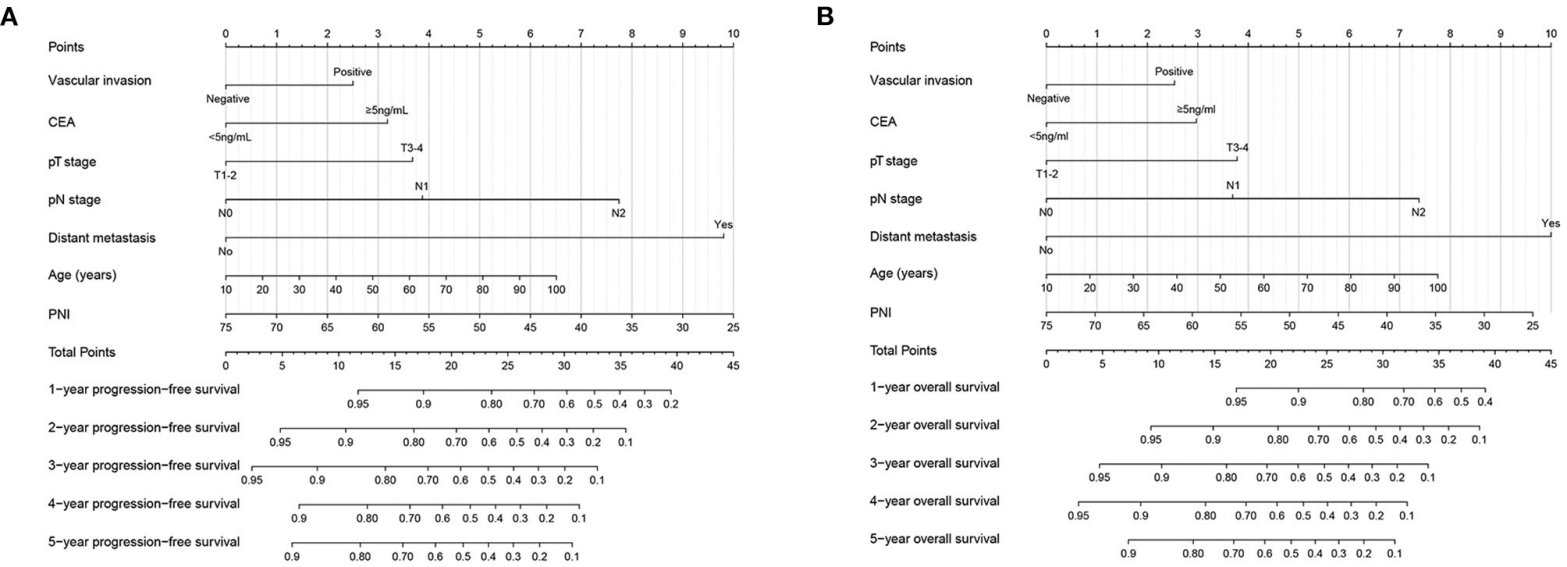
Construction the novel prognostic nomograms in CRC patients. **(A)** PFS nomogram; **(B)** OS nomogram.

The nomogram is considered a simple and effective tool to provide personalized risk prediction for patients. We developed a complication nomogram to predict the risk of postoperative complications in CRC patients (Figure 3A). The nomogram included operation time, intraoperative bleeding, age and PNI. The C-index of the complication nomogram was 0.646 (95%CI: 0.601–0.691), and the calibration curve showed a good consistency between the probability of complication predicted by the nomogram and the actual results (Figure 3B). These results indicated that our complication nomogram had good predictive accuracy in predicting postoperative complications in CRC patients. Similarly, based on prognostic variables identified in multivariate survival analysis (vascular invasion, CEA level, pT stage, pN stage, distant metastasis, age, and PNI), we developed two survival nomograms to predict 1–5-years PFS (Figure 4A) and OS (Figure 4B) of CRC patients. The C-index of PFS and OS nomogram was 0.723(95%CI: 0.712–0.735) and 0.729(95%CI: 0.705–0.753), and the calibration curves of PFS (Supplementary Figures S6A,B) and OS (Supplementary Figure S6C,D) all proved the best consistency between the predicted survival probability and the actual observed value. These results indicated that the prognostic nomogram we constructed had good predictive accuracy in predicting the prognosis of CRC patients.

We compared our nomograms with traditional TNM stage system through time-dependent ROC. The results showed that compared with TNM stage, our nomogram had better resolution and accuracy in predicting PFS (Supplementary Figures S7A,B) and OS (Supplementary Figures S7C,D) at 3- and 5- year. In addition, the DCA showed that when the threshold probability of predicting 3- and 5-year PFS was between 10 and 57% and 10 and 76%, respectively, the PFS nomogram showed a net benefit superior to the TNM stage system (Supplementary Figures S8A,B). Similar results also appeared on the OS nomogram, when the threshold probability of OS prediction at 3- and 5- year is 10–50% and 10–66%, respectively, the OS nomogram showed a net benefit superior to TNM stage (Supplementary Figures S8C,D). The above results indicated that compared with the traditional TNM stage system, the PNI-based nomograms could obtain higher net benefit within a larger threshold probability range.

## Discussion

Tumor inflammatory microenvironment plays an important role in cancer progression (14). Virchow et al. first detected the presence of tumor-infiltrating lymphocytes in 1,881 and speculated that the occurrence of tumors might be related to inflammation (15). Hanahan et al. (16) further found that immune and inflammatory cells were an important part of the tumor microenvironment, they could produce cytokines and chemokines through autocrine and paracrine ways to influence tumor growth. Recently, a variety of prognostic indicators based on cancer-related inflammation have been developed to predict surgical risk and tumor prognosis (17, 18). PNI, established by Onodera et al. (19), is a simple and easy parameter to reflect the immune and inflammatory status and has been proved to be an effective prognostic indicator for various malignancy (7–9). The latest research suggested that serum albumin was associated with systemic inflammation. The decrease in serum albumin may be the result of the combined effect of the liver reordering of protein synthesis in the body under high inflammation and the redistribution of albumin inside and outside blood vessels (20). In addition, hypoalbuminemia reflects malnutrition and impaired immune response of patients, which is associated with increased disease severity, high risk of progression, and low survival (21, 22). Lymphocytes play a vital role in cancer immune monitoring, which can inhibit the proliferation and growth of tumor cells by mediating cytotoxic cell death (6). It has been reported that a low peripheral lymphocyte may indicate an inadequate immune response to tumor, which will create a favorable microenvironment for tumor recurrence and lead to poor prognosis (23). Therefore, the PNI can reflect not only the nutritional status of patients, but also the cancer-related immune inflammatory response.

In this study, we demonstrated that preoperative PNI was a useful predictor of postoperative complications and long-term outcome in CRC patients. We found that low PNI was associated with high tumor burden, invasive pathological features, and poor host status, which was consistent with a number of previous studies (10, 24, 25). Notably, PNI was significantly associated with CEA and TNM stage, suggesting that PNI has similar prognostic value to CEA and TNM stage. That is, PNI calculated based on routine preoperative laboratory data has great potential as a predictor of CRC invasion potential. In addition, we found that PNI was superior to conventional prognostic indicators of inflammation in predicting the prognosis of CRC patients. Malnutrition and hyper-inflammatory status increase the risk of postoperative complications in CRC patients. In our study, approximately 17.8% of CRC patients had postoperative complications of varying degrees. The rate of postoperative complications in the low PNI group reached 24.6%, while that of the high PNI group was only 14.4%. Thus, patients with low PNI were more prone to postoperative complications. In addition, multivariate analysis showed that low PNI was an independent risk factor for postoperative complications in CRC patients. Studies have shown that postoperative complications have a negative impact on the survival of patients, and the severity of complications is related to the survival time of patients with malignancy (26, 27). This may be due to the complications enhance the systemic inflammatory response (28). Perioperative immunonutritional support can reduce postoperative complications in malnourished patients (29, 30). We constructed a simple and effective complication prediction nomogram based on risk factors identified in multivariate analysis, which can provide a scientific basis for the implementation of nutritional support.

The TNM staging system is currently recognized as the most effective tool for predicting disease progression and designing treatment strategies in CRC patients. However, it has been reported that patients with the same TNM stage may still have different clinical outcomes (31). In this study, PNI could effectively stratify the prognosis of CRC patients in each stage, which showed PNI could be used as a useful supplement to TNM stage. In addition, compared with early CRC patients, PNI could more effectively stratify the prognosis of advanced CRC patients. This may be related to the following reasons: advanced tumor have a higher tumor load and are more prone to proliferation, invasion, and neovascularization, leading to high systemic inflammation. In addition, patients with advanced tumors are prone to obstruction, bleeding, and reduced food intake, which leads to decreased nutritional status. In summary, we believed that preoperative PNI is a reliable, objective, reproducible and cheap predictive indicator for CRC patients undergoing surgical treatment, and can be considered as a routine clinical application.

For convenient and intuitive use in clinical practice, we constructed novel and effective prognostic nomograms for personalized prognostic evaluation in CRC patients. These nomograms have the advantage of integrating personal conditions, tumor characteristics, serum tumor markers and nutritional and immune inflammatory-related markers. Compared with TNM stage, the nomograms have better resolution and accuracy in predicting the 3- and 5-year PFS and OS of CRC patients. These nomograms can help to develop individualized risk stratification, individualized follow-up, and treatment strategies for CRC patients. These models can directly help clinicians quantify the prognostic risk of CRC patients, thus making it easier to formulate appropriate treatment strategies for CRC patients.

This study demonstrated that PNI was a useful indicator for predicting postoperative complications and long-term prognosis of CRC patients. Different from previous studies, we evaluated the prognostic value of PNI in CRC patients from multiple perspectives, including postoperative complications, hospital stay, PFS and OS, which provided a favorable reference for comprehensively evaluating the prognostic value and clinical application prospects of PNI in CRC patients. In addition, we have constructed PNI-based nomograms, which can be more personalized and convenient to use in clinical practice. In the era of precision medicine, individualized and specific management of patients is required. These analyses may provide further insights for the nutritional or immunological evaluation of CRC patients. However, there are some limitations to our study. First, this was a retrospective single-center study, so further validation of our results in a large sample, prospective cohort is needed in the future. In addition, since preoperative PNI was assessed only at a single time point, it failed to reflect the impact of PNI trajectory changes on prognosis, which requires further exploration in future studies. Finally, due to the limited samples, PNI-based nomograms could not be further validated. In the future, we expect to be able to further validate the accuracy of nomograms we constructed in large samples and multiple centers.

## Conclusion

This study demonstrated that PNI was an independent risk factor affecting postoperative complications, PFS and OS in CRC patients, and was a useful supplement to the TNM stage. PNI-based nomograms had good predictive accuracy and could be used to individually assess the prognosis of CRC patients.

## Data Availability Statement

The original contributions presented in the study are included in the article/Supplementary Material, further inquiries can be directed to the corresponding author.

## Ethics Statement

This research strictly complied with the provisions of the Helsinki Declaration and was approved by the institutional review boards of the participating institution.

## Author Contributions

JG conception and design. JG and ST management support. HX, GY, and ML data collection. HX data analysis and professional drafting. HX and LW manuscript writing. All authors agreed to publish. All authors contributed to the article and approved the submitted version.

## Conflict of Interest

The authors declare that the research was conducted in the absence of any commercial or financial relationships that could be construed as a potential conflict of interest.

## Publisher's Note

All claims expressed in this article are solely those of the authors and do not necessarily represent those of their affiliated organizations, or those of the publisher, the editors and the reviewers. Any product that may be evaluated in this article, or claim that may be made by its manufacturer, is not guaranteed or endorsed by the publisher.

## Supplementary Material

Supplementary Figure S1 The optimal cutoff value of PNI was determined by optimal stratification method.

Supplementary Figure S2 Box plot of clinicopathological features based on low and high PNI group. The boxplots show the 5 and 95% confidence intervals. The box plot lower extreme is the first quartile and the box plot upper extreme is the third quartile. Box plots show the median and whiskers are the minimum and maximum, respectively. The statistical method used for each group was the student's *t*-test. *p* > 0.05, ^*^*p* < 0.05, ^**^*p* < 0.01, ^***^*p* < 0.001.

Supplementary Figure S3 Postoperative complication rate and hospital stay according to preoperative prognostic nutritional index categories.

Supplementary Figure S4 Subgroup survival analysis based on various clinicopathological features. **(A)** Subgroup PFS analysis; **(B)** Subgroup OS analysis.

Supplementary Figure S5 Comparison of the ability of PNI and other prognostic indexes in predicting prognosis of CRC patients using ROC curves. **(A)** PFS at 3-year point; **(B)** PFS at 5-year point; **(C)** OS at 3-year point; **(D)** OS at 5-year point.

Supplementary Figure S6 Calibration curve of novel complication nomograms. **(A)** 3-year PFS; **(B)** 5-year PFS; **(C)** 3-year OS; **(D)** 5-year OS. The X axis presents the predicted probability and the Y axis shows the actual probability. The calibration lines fit along with the 45°reference.

Supplementary Figure S7 Comparison of the ability of novel prognostic nomograms and TNM classification in predicting prognosis at 3-year and 5-year point. **(A)** 3-year PFS; **(B)** 5-year PFS; **(C)** 3-year OS; **(D)** 5-year OS.

Supplementary Figure S8 Decision curve analyses of novel prognostic nomograms and TNM classification for **(A)** 3-year PFS; **(B)** 5-year PFS; **(C)** 3-year OS; and **(D)** 5-year OS.

Supplementary Table S1 Details of postoperative complications according to modified Clavien grading system.

Supplementary Table S2 Univariate and multivariate Logistic regression analysis of complications in CRC patients.

Supplementary Table S3 The association between prognostic nutritional index and hazard ratio of CRC patients.
