# Supplementary figures and images for: Prognostic Value of Prognostic Nutritional Index in Patients With Colorectal Cancer Undergoing Surgical Treatment
Source: Front Nutr. 2022 Mar 11;9:794489. doi: 10.3389/fnut.2022.794489 (PMC8963789; doi:10.3389/fnut.2022.794489)

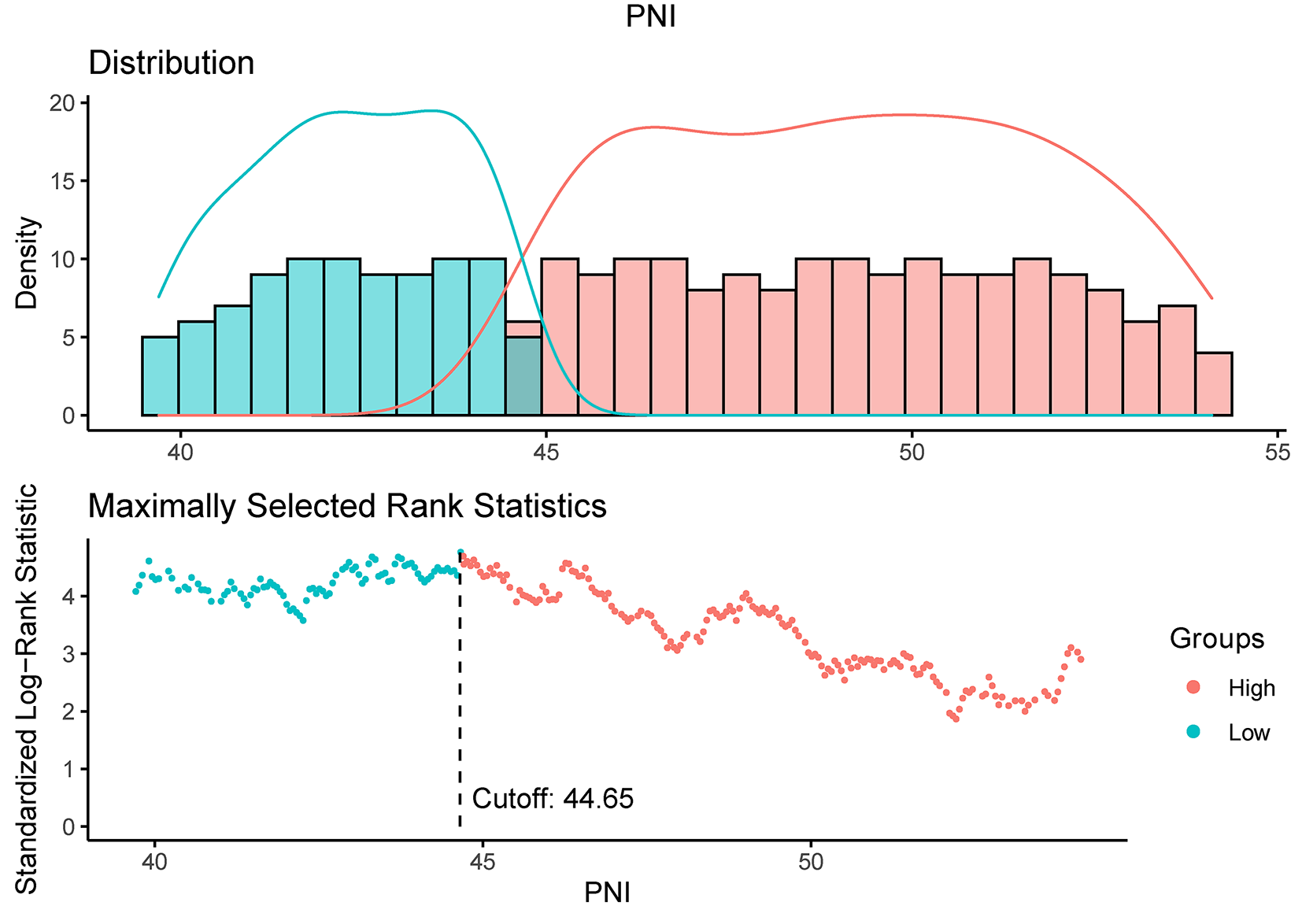

Supplement: Supplementary Figure S1 — The optimal cutoff value of PNI was determined by optimal stratification method. [file Image_1.TIF]

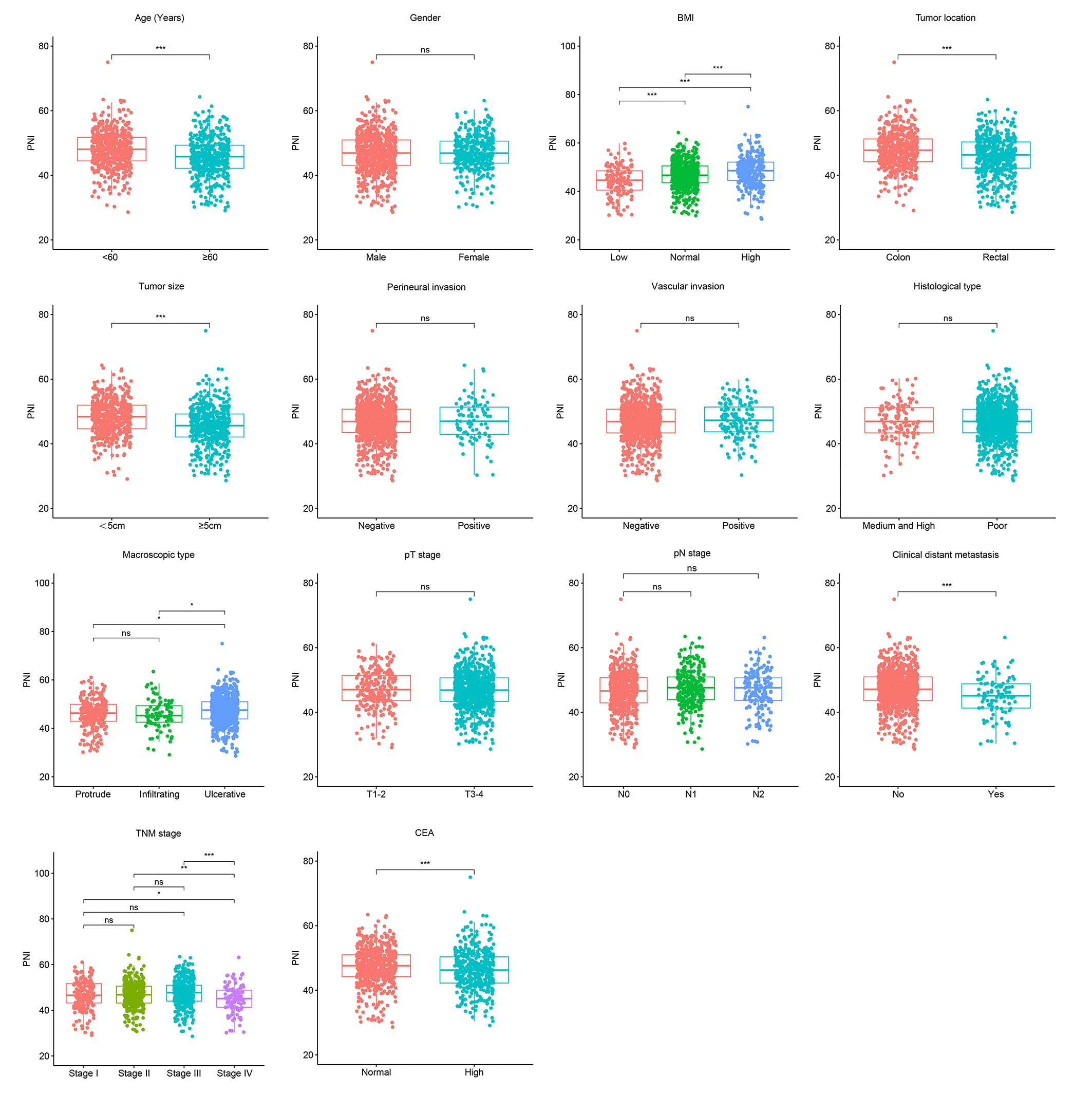

Supplement: Supplementary Figure S2 — Box plot of clinicopathological features based on low and high PNI group. The boxplots show the 5 and 95% confidence intervals. The box plot lower extreme is the first quartile and the box plot upper extreme is the third quartile. Box plots show the median and whiskers are the minimum and maximum, respectively. The statistical method used for each group was the student's t-test. p > 0.05, *p < 0.05, **p < 0.01, ***p < 0.001. [file Image_2.TIF]

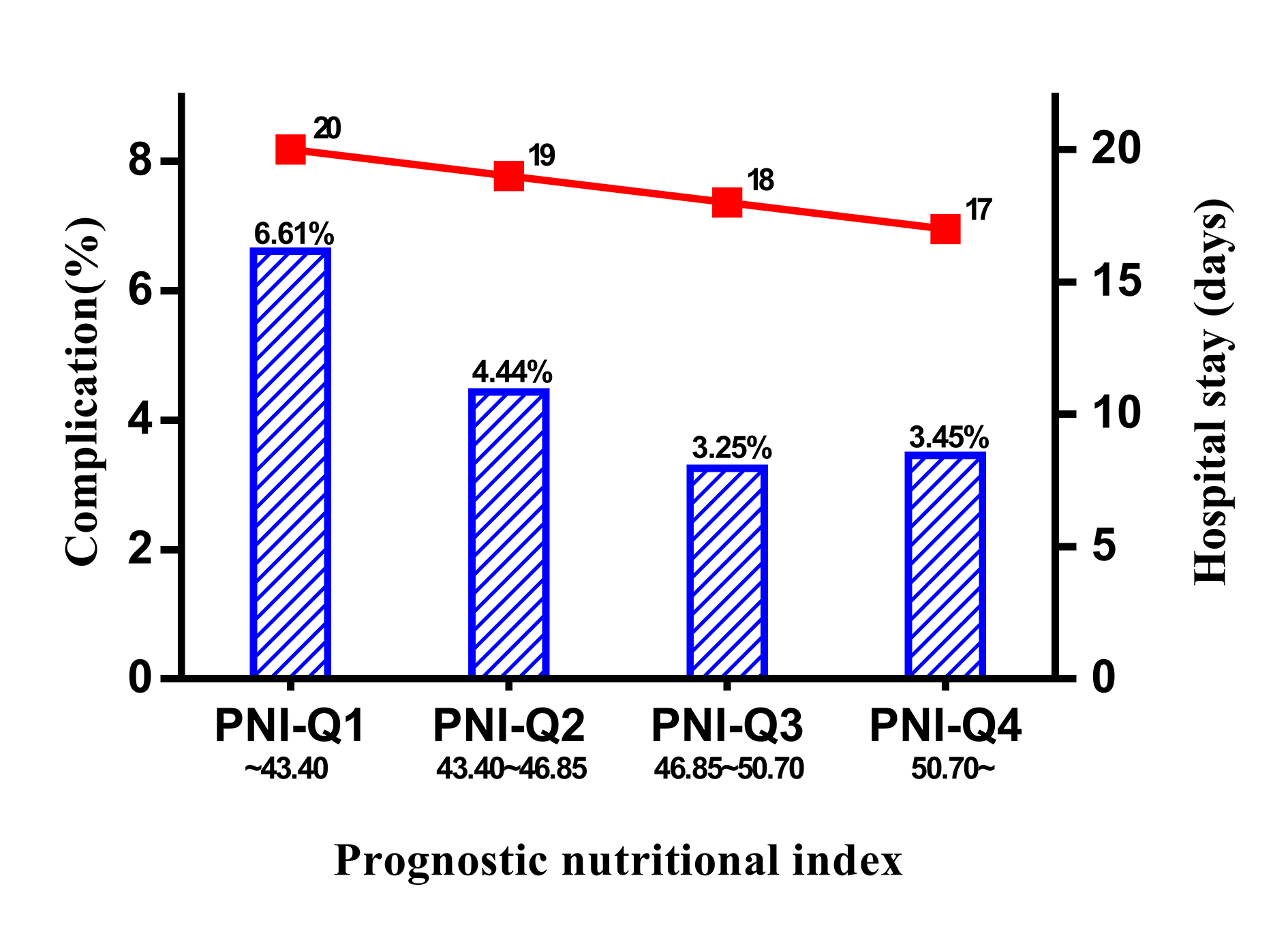

Supplement: Supplementary Figure S3 — Postoperative complication rate and hospital stay according to preoperative prognostic nutritional index categories. [file Image_3.TIF]

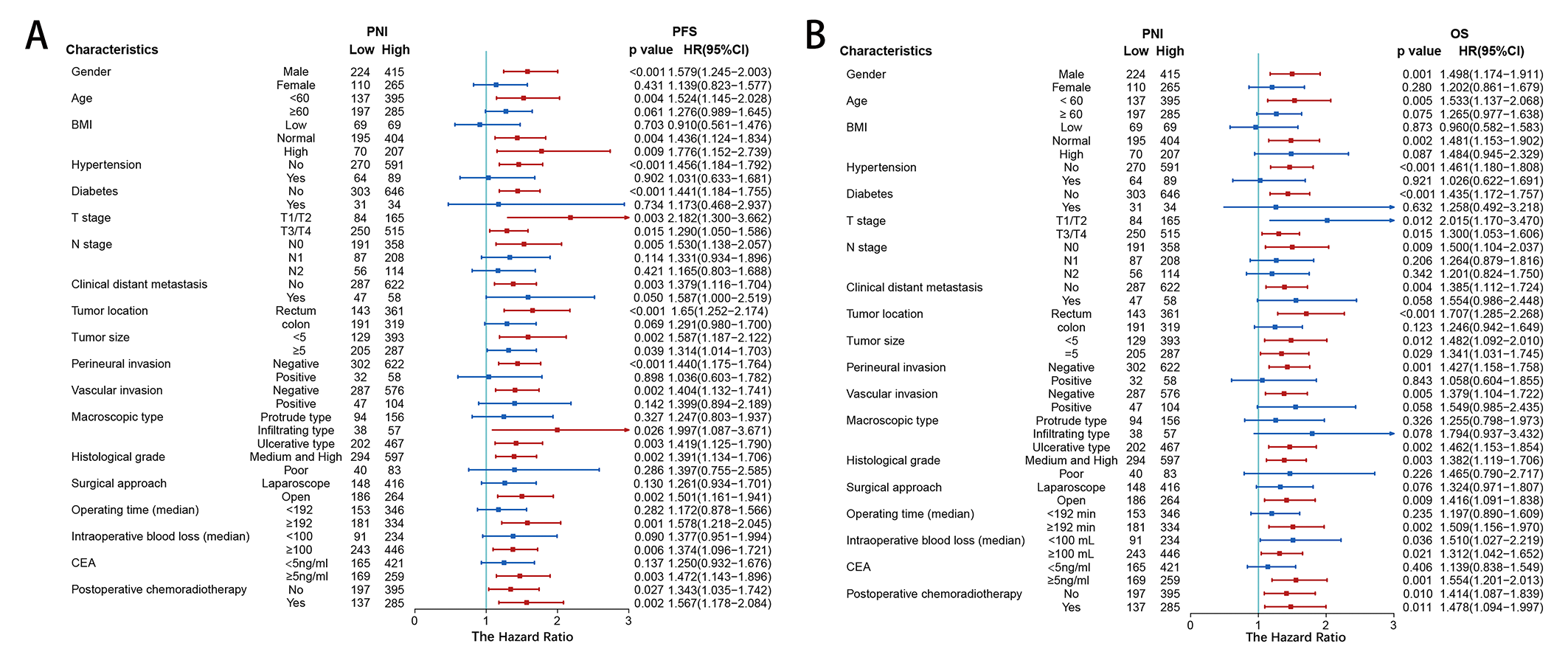

Supplement: Supplementary Figure S4 — Subgroup survival analysis based on various clinicopathological features. (A) Subgroup PFS analysis; (B) Subgroup OS analysis. [file Image_4.TIF]

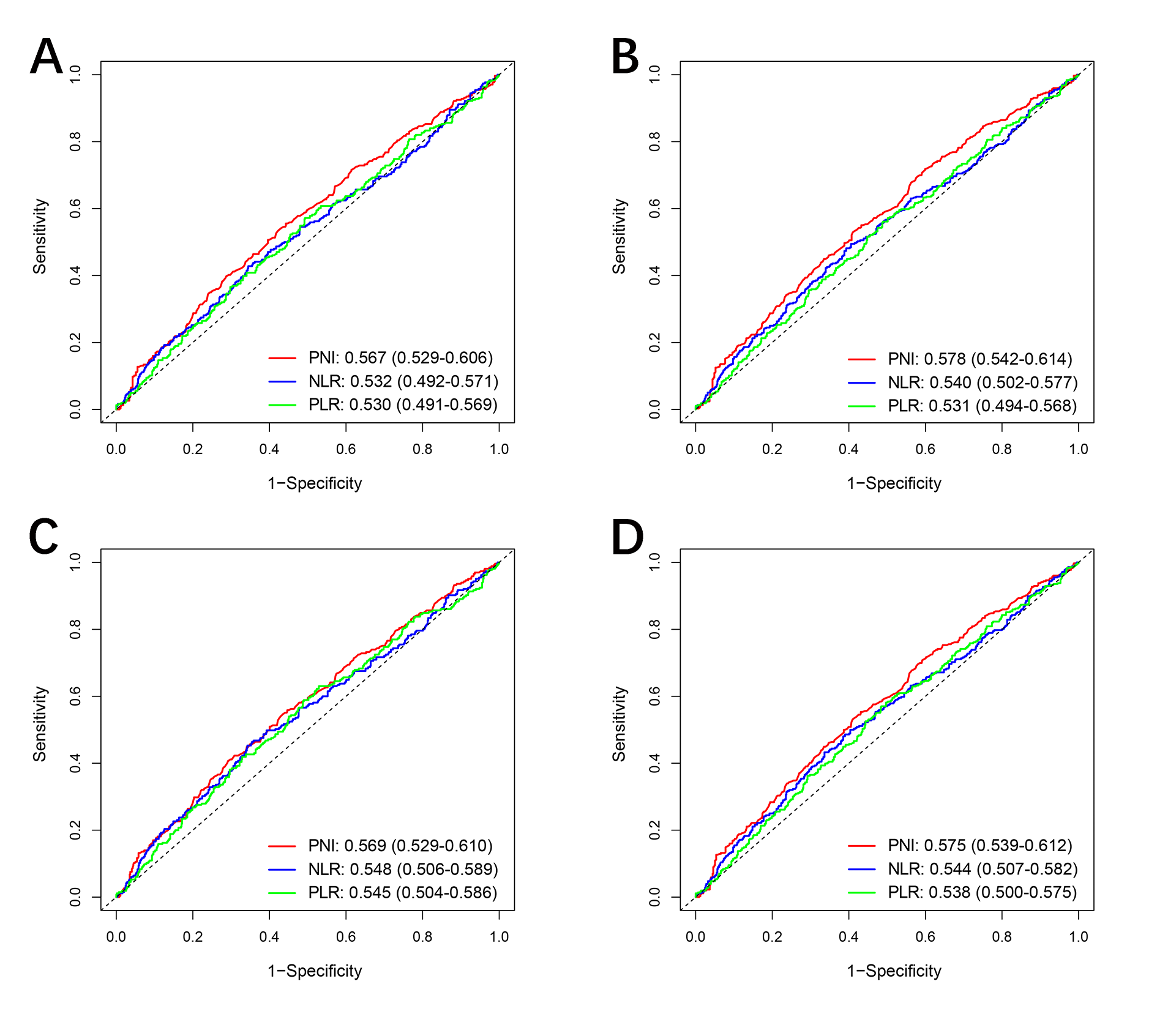

Supplement: Supplementary Figure S5 — Comparison of the ability of PNI and other prognostic indexes in predicting prognosis of CRC patients using ROC curves. (A) PFS at 3-year point; (B) PFS at 5-year point; (C) OS at 3-year point; (D) OS at 5-year point. [file Image_5.TIF]

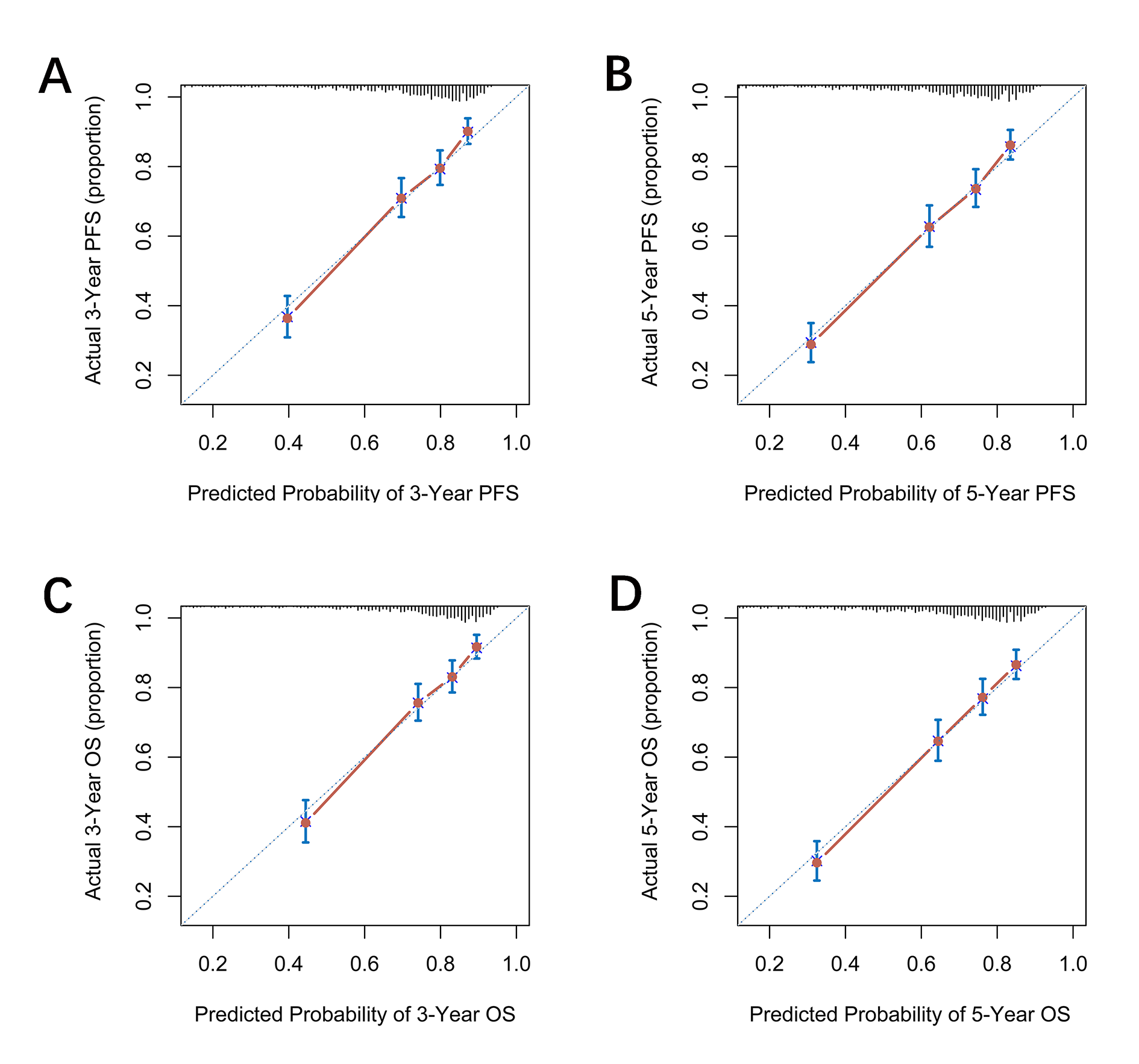

Supplement: Supplementary Figure S6 — Calibration curve of novel complication nomograms. (A) 3-year PFS; (B) 5-year PFS; (C) 3-year OS; (D) 5-year OS. The X axis presents the predicted probability and the Y axis shows the actual probability. The calibration lines fit along with the 45°reference. [file Image_6.TIF]

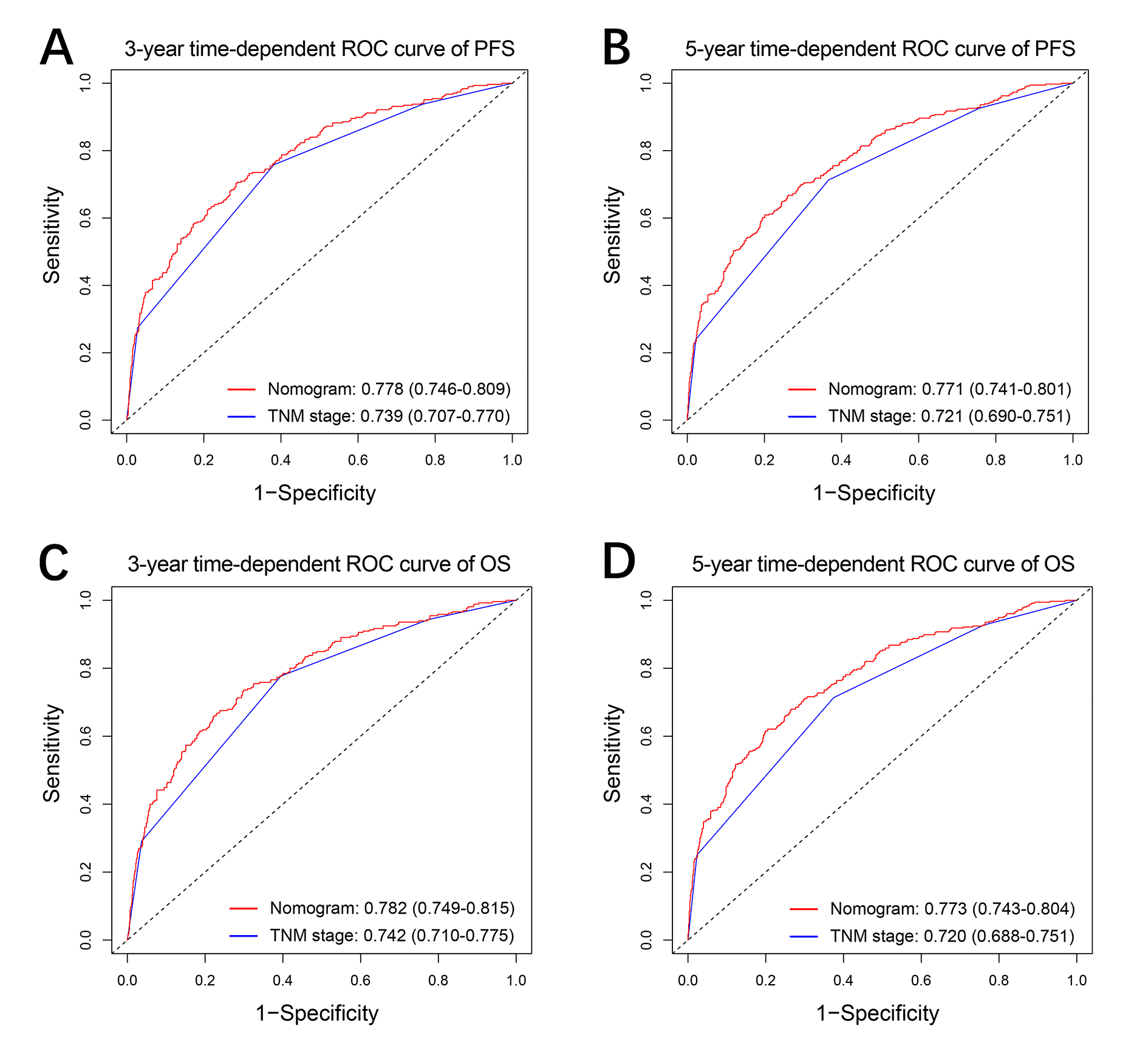

Supplement: Supplementary Figure S7 — Comparison of the ability of novel prognostic nomograms and TNM classification in predicting prognosis at 3-year and 5-year point. (A) 3-year PFS; (B) 5-year PFS; (C) 3-year OS; (D) 5-year OS. [file Image_7.TIF]

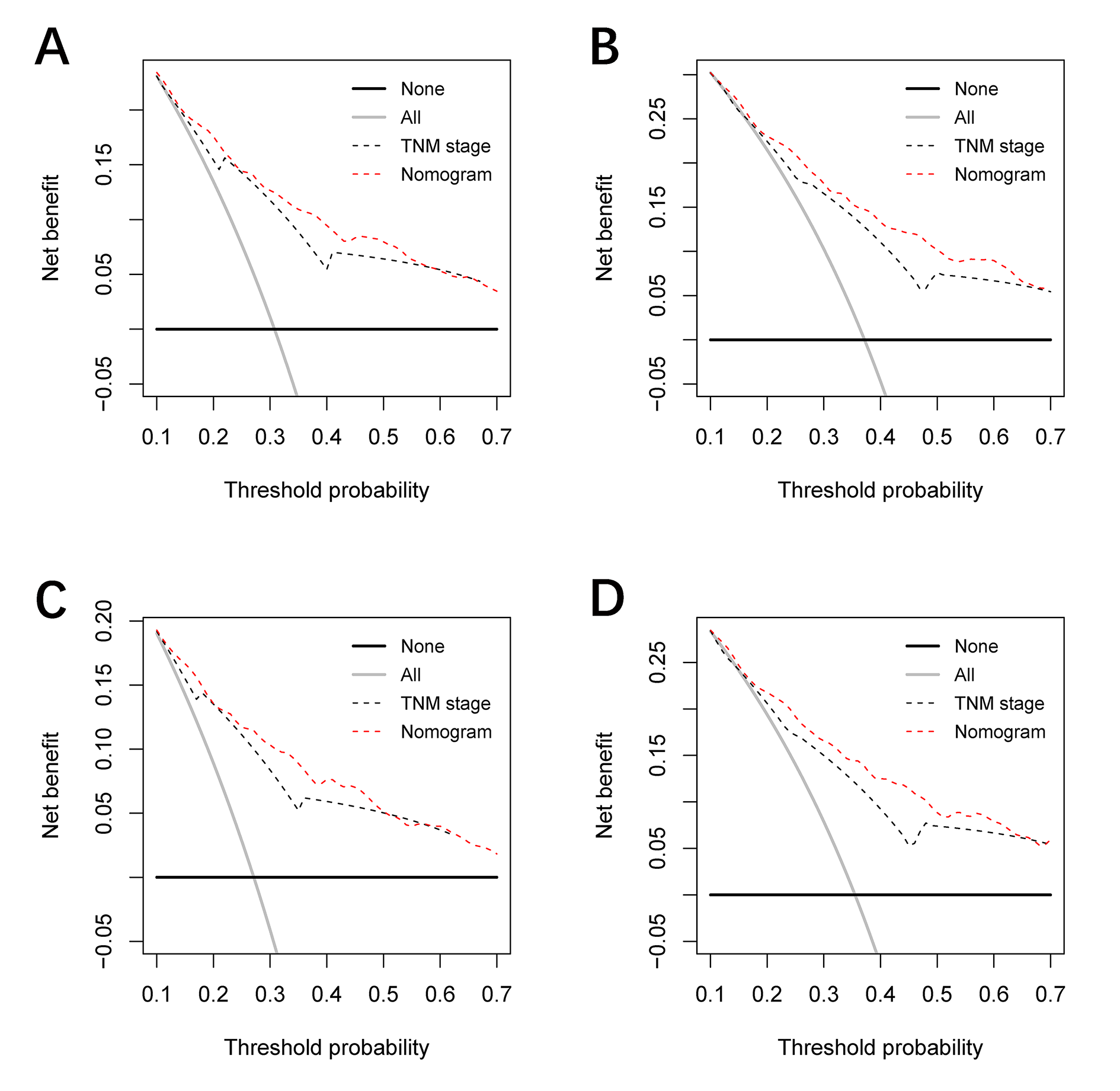

Supplement: Supplementary Figure S8 — Decision curve analyses of novel prognostic nomograms and TNM classification for (A) 3-year PFS; (B) 5-year PFS; (C) 3-year OS; and (D) 5-year OS. [file Image_8.TIF]
