# Supplementary material for: Prognostic Value of Prognostic Nutritional Index in Patients With Colorectal Cancer Undergoing Surgical Treatment
Source: Front Nutr. 2022 Mar 11;9:794489. doi: 10.3389/fnut.2022.794489 (PMC8963789; doi:10.3389/fnut.2022.794489)
Supplement: Supplementary Table S1 — Details of postoperative complications according to modified Clavien grading system. [file Table_1.DOCX]

**Table S1** Details of postoperative complications according to modified Clavien grading system.

| Grade | Total (n=1014) | PNI | | X^2^ | p value |
| --- | --- | --- | --- | --- | --- |
|  |  | Low (n = 334) | High (n = 680) |  |  |
| Total complications | 180(17.8%) | 82(24.6%) | 98(14.4%) | 15.771 | <0.001 |
| Grade I | 64(6.3%) | 32(9.6%) | 32(4.7%) | 9.002 | 0.003 |
| Grade II | 89(8.8%) | 34(10.2%) | 55(8.1%) | 1.224 | 0.269 |
| Grade III | 18(1.8%) | 10(2.3%) | 8(1.5%) | 4.244 | 0.039 |
| Grade IIIa | 10(1.0%) | 6(1.8%) | 4(0.6%) | 3.348 | 0.067 |
| Grade IIIb | 8(0.8%) | 4(1.2%) | 4(0.6%) | 1.063 | 0.303 |
| Grade IV | 8(0.8%) | 5(1.5%) | 3(0.4%) | 3.190 | 0.074 |
| Grade IVa | 5(0.5%) | 3(0.9%) | 2(0.3%) | 1.666 | 0.197 |
| Grade IVb | 3(0.3%) | 2(0.6%) | 1(0.1%) | 1.550 | 0.213 |
| Grade V | 1(0.1%) | 1(0.3%) | 0(0.0%) | 2.038 | 0.329 |
