# Supplementary material for: Prognostic Value of Prognostic Nutritional Index in Patients With Colorectal Cancer Undergoing Surgical Treatment
Source: Front Nutr. 2022 Mar 11;9:794489. doi: 10.3389/fnut.2022.794489 (PMC8963789; doi:10.3389/fnut.2022.794489)
Supplement: Supplementary Table S2 — Univariate and multivariate Logistic regression analysis of complications in CRC patients. [file Table_2.DOCX]

Table S2 Univariate and multivariate Logistic regression analysis of complications in CRC patients.

| **Characteristic** | **Complication** | | | |
| --- | --- | --- | --- | --- |
|  | Univariate analysis | | Multivariate analysis | |
|  | HR (95%CI) | P value | HR (95%CI) | P value |
| Gender (Female) | 1.178 (0.839, 1.653) | 0.343 |  |  |
| Age (≥60 years) | 1.897 (1.366, 2.636) | <0.001 | 1.677 (1.181, 2.380) | 0.004 |
| BMI |  | 0.631 |  |  |
| Low | Ref. |  |  |  |
| Normal | 1.028 (0.636, 1.661) | 0.910 |  |  |
| High | 0.854 (0.497, 1.465) | 0.565 |  |  |
| Hypertension (Yes) | 1.673 (1.112, 2.517) | 0.014 | 1.284 (0.831, 1.982) | 0.260 |
| Diabetes (Yes) | 1.563 (0.867, 2.816) | 0.137 |  |  |
| PNI (Low) | 1.932 (1.391, 2.684) | <0.001 | 1.580 (1.122, 2.27) | 0.009 |
| pT stage (T3-4) | 1.217 (0.826, 1.793) | 0.321 |  |  |
| pN stage |  | 0.584 |  |  |
| N0 | Ref. |  |  |  |
| N1 | 1.185 (0.824, 1.703) | 0.360 |  |  |
| N2 | 0.954 (0.601, 1.515) | 0.843 |  |  |
| Distant metastasis (Yes) | 1.180 (0.718, 1.942) | 0.514 |  |  |
| Tumor location (Colon) | 0.838 (0.607, 1.157) | 0.731 |  |  |
| Tumor size (≥5cm) | 1.134 (0.822, 1.566) | 0.443 |  |  |
| Perineural invasion (Positive) | 1.364 (0.807, 2.307) | 0.246 |  |  |
| Vascular invasion (Positive) | 1.121 (0.721, 1.743) | 0.612 |  |  |
| Macroscopic type |  | 0.208 |  |  |
| Protrude type | Ref. |  |  |  |
| Infiltrating type | 0.995 (0.511, 1.937) | 0.988 |  |  |
| Ulcerative type | 1.375 (0.923, 2.048) | 0.117 |  |  |
| Histological grade (Poor) | 1.211 (0.756, 1.942) | 0.426 |  |  |
| Surgical approach (Open) | 1.637 (1.184, 2.263) | 0.003 | 1.314 (0.935, 1.847) | 0.115 |
| Operating time (median) (≥192 min) | 1.530 (1.104, 2.122) | 0.011 | 1.418 (1.009, 1.993) | 0.044 |
| Intraoperative bleeding (median) (≥100 mL) | 1.958 (1.336, 2.869) | 0.001 | 1.660 (1.109, 2.484) | 0.014 |
| CEA (≥5ng/ml) | 1.428 (1.034, 1.973) | 0.031 | 1.194 (0.854, 1.670) | 0.300 |

Table Note: CRC, colorectal cancer; BMI, body mass index; PNI, prognostic nutrition index.
