# Supplementary material for: Prognostic Value of Prognostic Nutritional Index in Patients With Colorectal Cancer Undergoing Surgical Treatment
Source: Front Nutr. 2022 Mar 11;9:794489. doi: 10.3389/fnut.2022.794489 (PMC8963789; doi:10.3389/fnut.2022.794489)
Supplement: Supplementary Table S3 — The association between prognostic nutritional index and hazard ratio of CRC patients. [file Table_3.DOCX]

Table S3. The association between prognostic nutritional index and hazard ratio of CRC patients

| PNI | Progression-free survival | | | | Overall survival | | | |
| --- | --- | --- | --- | --- | --- | --- | --- | --- |
|  | Crude HR (95%CI) | *p* | Adjusted HR (95%CI) | *p* | Crude HR (95%CI) | *p* | Adjusted HR (95%CI) | *p* |
| As continuous (per SD) | 0.964(0.950,0.979) | <0.001 | 0.977(0.962,0.992) | 0.003 | 0.963(0.948,0.978) | <0.001 | 0.977(0.962,0.993) | 0.004 |
| Binaries |  |  |  |  |  |  |  |  |
| B1 (~44.65) | ref |  | ref |  | ref |  | ref |  |
| B2 (44.65~) | 0.658(0.547,0.791) | <0.001 | 0.717(0.594,0.867) | 0.001 | 0.655(0.542,0.792) | <0.001 | 0.721(0.593,0.876) | 0.001 |
| p for trend |  | <0.001 |  |  |  | <0.001 |  |  |
| Tertiles |  |  |  |  |  |  |  |  |
| T1 ( ~44.65) | ref |  | ref |  | ref |  | ref |  |
| T2 ( 44.65~49.30) | 0.704(0.568,0.872) | 0.001 | 0.717(0.576,0.893) | 0.003 | 0.706(0.567,0.879) | 0.002 | 0.729(0.582,0.913) | 0.006 |
| T3 ( 49.30~) | 0.584(0.467,0.731) | <0.001 | 0.679(0.539,0.855) | 0.001 | 0.574(0.456,0.723) | <0.001 | 0.672(0.530,0.852) | 0.001 |
| p for trend |  | <0.001 |  | 0.001 |  | <0.001 |  | 0.001 |
| Quartiles |  |  |  |  |  |  |  |  |
| Q1 ( ~43.40) | ref |  | ref |  | ref |  | ref |  |
| Q2 (43.40~46.85) | 0.690(0.542,0.879) | 0.003 | 0.768(0.600,0.984) | 0.037 | 0.711(0.556,0.911) | <0.001 | 0.791(0.615,1.018) | 0.069 |
| Q3 (46.85~50.70) | 0.645(0.504,0.826) | 0.001 | 0.701(0.543,0.903) | 0.006 | 0.613(0.474,0.793) | 0.007 | 0.674(0.517,0.877) | 0.003 |
| Q4 (50.70~) | 0.539(0.417,0.698) | <0.001 | 0.651(0.499,0.848) | 0.001 | 0.552(0.424,0.719) | <0.001 | 0.666(0.509,0.873) | 0.003 |
| p for trend |  | <0.001 |  | 0.005 |  | <0.001 |  | 0.007 |

Table Note: CRC, colorectal cancer; BMI, body mass index; PNI, prognostic nutrition index.

Adjusted for gender, age, BMI, hypertension, diabetes, pT stage, pN stage, clinical distant metastasis, tumor location, tumor size, perineural invasion, vascular invasion, macroscopic type, histological type.
